# Effects of Cadmium on the Accumulation and Phytotoxicity of Uranium in Radish (*Raphanus sativus* L.) Seedlings

**DOI:** 10.3390/plants14172711

**Published:** 2025-09-01

**Authors:** Xin-Peng Guo, Xi Chen, Chun-Xia Tu, Yu-Meng Fan, Ming-Xuan Wang, Zheng-Qin Zhao, Shi-Yi Yang, Lan-Lan Cui, Guo Wu, Jin-Long Lai, Qun Li

**Affiliations:** 1 College of Life Science, Sichuan Normal University, Chengdu 610101, China; shixinpu19610613@163.com (X.-P.G.); 18328640034@163.com (Y.-M.F.); 13994652071@163.com (M.-X.W.); 13778309360@163.com (Z.-Q.Z.); 15328972277@163.com (S.-Y.Y.); cuiyounian@gmail.com (L.-L.C.); wuguoyk@sicnu.edu.cn (G.W.); 2 College of Resources and Environmental Sciences, Nanjing Agricultural University, Nanjing 210095, China; chenx_19981008@163.com; 3 State Key Laboratory of Cellular Stress Biology, Xiamen University, Xiamen 361000, China; tuchunxia@stu.xmu.edu.cn; 4 Engineering Research Center of Biomass Materials, Ministry of Education, Southwest University of Science and Technology, Mianyang 621010, China

**Keywords:** uranium, cadmium, *Raphanus sativus*, phytoremediation, phytotoxicity

## Abstract

Cadmium (Cd) is a major co-occurring, highly toxic heavy metal in uranium (U) tailings that poses synergistic risks to ecological and human health. This study aimed to investigate the effects of Cd on U accumulation and phytotoxicity in plants using radish (*Raphanus sativus* L.) as a model organism under hydroponic conditions. Treatments included U alone (25 μM and 50 μM), low-concentration Cd alone (10 μM), and U + Cd co-treatments (U25 + Cd and U50 + Cd). Results revealed that exposure exerted minimal phytotoxicity, whereas U treatment induced severe root toxicity, characterized by cell death and an 11.9–63.8% reduction in root biomass compared to the control. Notably, U + Cd co-treatment exacerbated root cell death and biomass loss relative to U alone. Physiologically, elevated U concentrations significantly increased superoxide anion radical (O_2_^−^) production rate, hydrogen peroxide (H_2_O_2_) content, and malondialdehyde (MDA)—a marker of oxidative damage—inducing cellular oxidative stress. Under U + Cd co-treatment, O_2_^−^ production, H_2_O_2_ content, and MDA levels in radish roots were all significantly higher than under U alone. Concurrently, activities of antioxidant enzymes (superoxide dismutase [SOD], catalase [CAT], and peroxidase [POD]) were lower in U + Cd-treated roots than in U-treated roots, further exacerbating oxidative damage. Regarding heavy metal accumulation, the content of U in radish under U + Cd treatment was significantly higher than that in the U treatment group. However, no significant differences were observed in the expression of uranium (U)-related transport genes (*MCA1*, *MCA3*, and *ANN1*) between the single U treatment and the U-Cd co-treatment. Notably, the inhibitory effect of *NRAMP3*—a gene associated with Cd transport—was weakened under the coexistence of U, indicating that U exacerbates toxicity by promoting Cd transport. This study shows that Cd appears to enhance the accumulation of U in radish roots and exacerbate the phytotoxicity of U.

## 1. Introduction

The advancement of nuclear energy has spurred extensive uranium (U) mining activities and subsequently resulted in serious soil and water contamination [1]. Uranium is a natural radionuclide, and U contamination is characterized by high toxicity, persistence, non-degradability, etc. [2]. In addition, U tailings also contain a variety of associated heavy metals, such as cadmium (Cd), lead (Pb), copper (Cu), manganese (Mn), and iron (Fe) [3]. According to one investigation, the most severe contamination in the soil surrounding the uranium mining is attributed to uranium and cadmium, with average concentrations of 39.88 mg/kg for uranium and 55.33 mg/kg for cadmium [4]. In conventional agricultural production, U and Cd are frequently detected simultaneously, with U accumulation increasing annually [5]. After being absorbed by plants, U and Cd not only imperil the development of crops but may also infiltrate the human body via the food chain, thereby posing a significant risk to human health [6]. Consequently, it is crucial to undertake comprehensive research focused on remediating the soil and water environments surrounding uranium tailings.

The remediation of soil contaminated with heavy metals primarily encompasses physical, chemical, and biological methods. Notably, phytoremediation technology has emerged as a focal point of research in recent years, attributed to its cost-effectiveness and eco-friendly attributes [7,8,9]. However, U and Cd are non-essential elements for plants, and their excessive accumulation can inhibit plant growth, consequently resulting in a significant decrease in remediation efficiency [10]. The effects of U and Cd stress on plant physiology and biochemistry have been extensively reported, including the following four aspects: (1) destroying the root microstructure and inhibiting its growth and development [6,11,12]; (2) inhibiting the antioxidant enzyme systems in plants and causing peroxidative damage [13,14,15,16]; (3) interfering with the metabolism of mineral elements (Fe, Mn, etc.) in plants [17,18,19]; and (4) decreasing the capacity for photosynthesis by interfering with chlorophyll metabolism and destroying the structure of the thylakoid [20,21,22,23].

The majority of pertinent studies have solely focused on individual uranium or cadmium stress. While several studies have explored the combined effects of uranium and cadmium to examine the interaction of their toxicities, the findings from different studies appear to be contradictory. For instance, previous studies have demonstrated that the presence of cadmium amplifies the toxicity of uranium in *Arabidopsis thaliana* and *Oenanthe javanica*, leading to a more pronounced inhibition of fresh weight, plant height, and root length [24,25]. On the contrary, the presence of Cd mitigated the toxic effects of U in *Rohdea japonica* [26]. More critically, while existing studies have identified several transporter proteins involved in U and Cd uptake, such as *Nramp*, *MCA*, *HMA*, and the *ANN1* protein family [27,28], their expression dynamics under combined U-Cd stress remain undiscovered. Therefore, further research is warranted to investigate the interaction of uranium and cadmium toxicity in plants.

Radish (*Raphanus sativus* L.), a member of the Brassicaceae family which exhibits robust phytoextraction capacity and tolerance for both U and Cd, is one of the richest in metal tolerant species [29,30]. Studies have demonstrated that radishes possess a remarkable and extensive capacity to enrich uranium or cadmium present in both soil and water [31,32,33,34,35]. Furthermore, radish offers additional experimental advantages: rapid growth and cost-effectiveness. Crucially, the large size of the main root of radishes serves to enhance their biogeochemical accumulation efficiency for heavy metals [36]. This unique property enables radish to effectively mitigate uranium and cadmium pollution in the environment, playing a crucial role in environmental remediation efforts [32,37,38,39]. Although the effects of individual U or Cd stress on the physiological metabolism of radish have been reported [40], it remains unclear whether the presence of Cd influences the accumulation and toxicity of U in radish. Therefore, in the present study, the accumulation and physiological characteristics of these two heavy metals in radish seedlings under single U, Cd, and U + Cd combined treatments are discussed. In addition, to verify the expression of the transporters between U and Cd, we selected six key transporters for gene expression analysis: *ANN1*, *NRAMP1*, *NRAMP3*, *HMA2*, *MCA1*, and *MCA3*. Our hypothesis suggests that the presence of cadmium would result in more severe uranium poisoning in radishes.

## 2. Results

### 2.1. Phytotoxicity of U and Cd in Radish Seedlings

**Figure 1:**
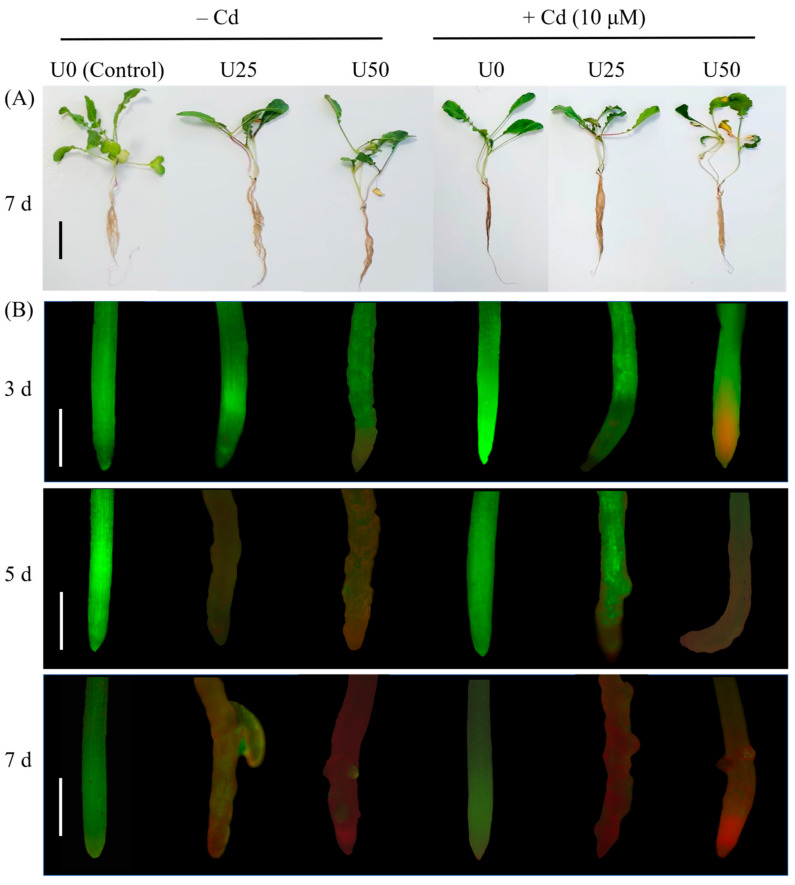
The phenotype and root activity of radish seedlings subjected to U treatment and U + Cd combined exposure. (**A**) The phenotype of radishes after undergoing 7 days of U and U + Cd treatment. Scale bars represent 5 cm. (**B**) Root viability of radish after 3, 5, and 7 days of U and U + Cd treatment. Viable cells were stained with FDA, resulting in green fluorescence, while non-viable cells (with compromised plasma membranes) were stained with PI and exhibited red fluorescence (50× magnification). Scale bars represent 0.5 mm. +Cd denotes the treatment in the presence of Cd. −Cd indicates the treatment in the absence of Cd.

After single and combined treatments with U and Cd for a duration of 7 days, the growth of radish seedlings was significantly impeded in comparison to the control group (Figure 1A). The outcomes derived from FDA/PI staining revealed that the quantity of dead cells in the radish roots increased markedly in correlation with both the concentration of exogenous uranium and the length of exposure to the treatment. Following a treatment period of 7 days, the treatment in the presence of cadmium (+Cd) exhibited a notably higher number of dead cells than the treatment in the absence of cadmium (−Cd) (Figure 1B). Furthermore, a single cadmium treatment did not result in substantial cell death within the radish. These observations imply that the subdued concentration of cadmium present in uranium tailings exerts minimal toxic effects on radishes; nevertheless, the co-occurrence of Cd amplifies the toxic impacts of U in these plants.

**Figure 2:**
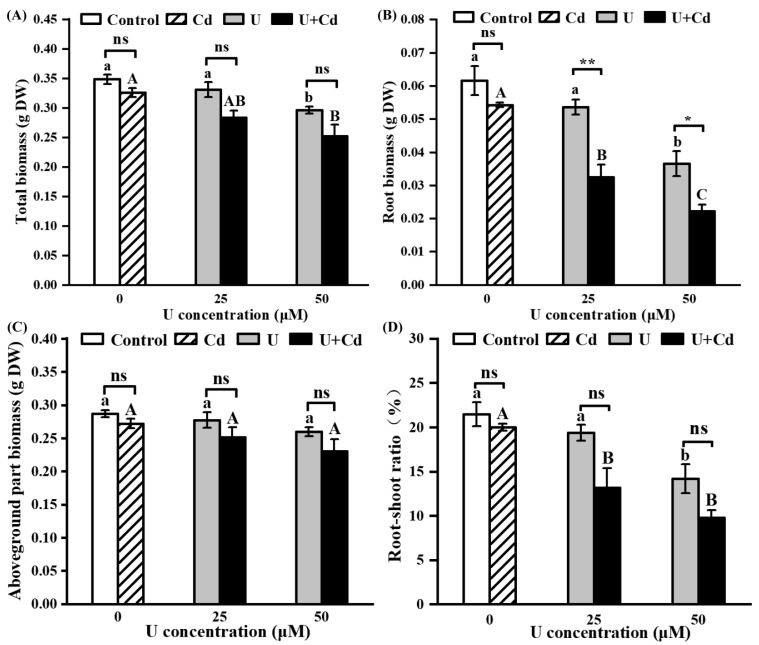
Biomass and root–shoot ratio of uranium (U) and combined uranium and cadmium (U + Cd) stress. (**A**) Root biomass, (**B**) aboveground part biomass, (**C**) total biomass, (**D**) root–shoot ratio. Data are presented as the mean ± SD of three independent biological replicates. The cadmium (Cd) treatment concentration was 10 μM. Distinct lowercase letters (a, b, etc.) denote significant differences between treatments with varying uranium (U) concentrations in the absence of Cd at the 0.05 level, while distinct uppercase letters (A, B, C, etc.) signify significant differences between different U concentrations in the presence of Cd at the 0.05 level (ANOVA and LSD multiple comparison). Asterisks * and ** indicate significant differences at the 0.05 and 0.01 levels, respectively, between treatments without Cd (−Cd) and with Cd (+Cd) at the same U concentration (Student’s *t*-test). n.s., nonsignificant.

After Cd treatment, the biomass and root–shoot ratio of radish had no significant change compared with the control group, suggesting that a low concentration of Cd exerts minimal toxicity on the radish. After U treatments, the biomass per radish plant diminished by 5% to 27.5% compared to the control group (Figure 2A). Notably, the root biomass experienced a more pronounced decrease, ranging between 11.9% and 63.8%, while the aboveground biomass remained relatively unaffected (Figure 2B,C). This disproportionate reduction led to a significantly lower root–shoot ratio compared to the control (Figure 2D), underscoring that uranium predominantly exerts its toxic effects on radish roots.

### 2.2. U and Cd Accumulation in Radish Seedlings

**Figure 3:**
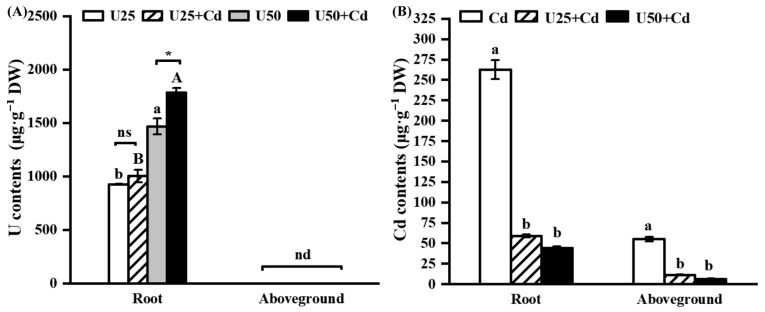
Enrichment characteristics of U and Cd in radish seedlings after 7 days of U and U + Cd stress. (**A**) U content. Different lowercase letters (a, b, etc.) indicate significant differences between treatments with different U concentrations at the 0.05 level (−Cd), and different uppercase letters (A, B, etc.) indicate significant differences between different U concentrations at the 0.05 level (+Cd). “*” indicates significant differences at the 0.05 levels between −Cd and +Cd treatments at the same U concentration, respectively (Student’s *t*-test). n.s., nonsignificant. nd, not detected. (**B**) Cd contents. Different lowercase letters (a, b, etc.) indicate significant differences between different U concentration treatments in the same organ at the 0.05 level (ANOVA and LSD multiple comparison). Data shown are mean ± SD of three independent biological replicates.

After U treatment and U + Cd combined treatment, both the single U and the U + Cd combined treatments led to an increase in the U content within the radish roots, which directly correlated with the escalation of exogenous U concentration. Notably, no traces of U were detected in the aboveground portions of the radishes. Under U + Cd treatment, the uranium content in the radish roots was higher than that under the single U treatment. Furthermore, at an exogenous uranium concentration of 50 μM, the uranium content in the roots subjected to the U + Cd treatment was 1.2 times greater than that in the roots exposed to the single U treatment (Figure 3A). Compared to the Cd treatment, the Cd content in both the roots and the aboveground parts of the radish was significantly reduced after the U + Cd treatment, decreasing by 77.6% to 83.3% and 79.8% to 88.3%, respectively, compared to the single Cd treatment (Figure 3B). These results suggest that in uranium tailings, where U and Cd coexist, there is an elevated accumulation of U in the roots of the radish, while conversely, there is a diminished accumulation of Cd throughout the plant.

### 2.3. U and Cd Interfere with the ROS Metabolism in Radish Roots

**Figure 4:**
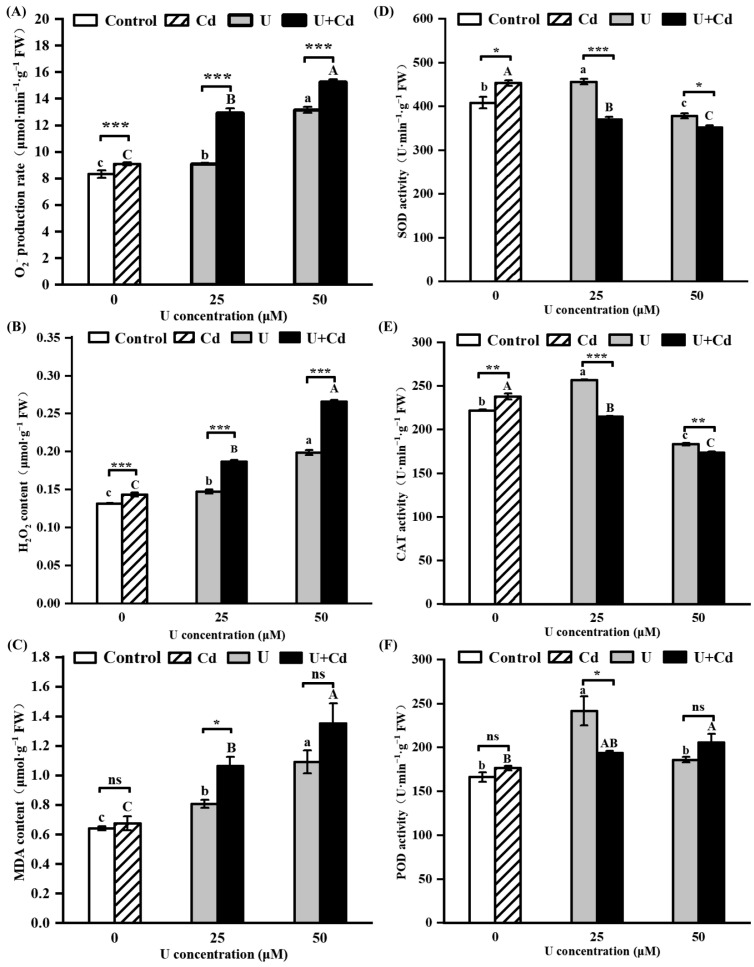
The ROS metabolism of radish roots after 7 days of U and U + Cd stress. (**A**) Superoxide anion (O_2_^−^) production rate, (**B**) hydrogen peroxide (H_2_O_2_) content, (**C**) malondialdehyde (MDA) content, (**D**) superoxide dismutase (SOD) activity, (**E**) catalase (CAT) activity, (**F**) peroxidase (POD) activity. Data shown are mean ± SD of three independent biological replicates. Different lowercase letters (a, b, c, etc.) indicate significant differences between treatments with different U concentrations at the 0.05 level (−Cd), and different uppercase letters (A, B, C, etc.) indicate significant differences between different U concentrations at the 0.05 level (+Cd) (ANOVA and LSD multiple comparison). “*”, “**” and “***” indicate significant differences at the 0.05, 0.01, and 0.001 levels between −Cd and +Cd treatments at the same U concentration, respectively (Student’s *t*-test). n.s., nonsignificant.

From phenotypic and biomass perspectives, U treatment and U + Cd combined treatment predominantly demonstrated toxic effects on the radish roots. We conducted an assessment of the reactive oxygen species burst and the activity levels of antioxidant enzymes within radish roots to determine if the presence of Cd intensifies the oxidative stress induced by U in radish. In the case of single Cd treatment, the production rate of O_2_^−^ and the content of H_2_O_2_ in the radish roots were notably elevated compared to the control group (Figure 4A,B). However, the content of MDA remained statistically similar to the control, suggesting that exposure to a low concentration of Cd led to an increase in reactive oxygen species in radish root cells without resulting in significant oxidative cell damage (Figure 4C). In both the U treatment and the U + Cd combined treatment, the rate of O_2_^−^ production (Figure 4A), H_2_O_2_ content (Figure 4B), and MDA content (Figure 4C) in the radish roots all increased significantly with the increase in U concentration. Specifically, these values were elevated by 1.1 to 1.8 times, 1.1 to 2.0 times, and 1.1 to 2.1 times the control group, respectively. This indicates that both single U treatment and U + Cd combined treatment caused oxidative damage to the cells. Further findings showed that after U + Cd combined treatment, the rate of O_2_^−^ production, H_2_O_2_ content, and MDA content in radish roots were all higher than those in the single U treatment (Figure 4A–C), indicating that the presence of Cd makes the oxidative stress induced by uranium stress in radish roots more severe.

Antioxidant enzymes, including SOD, CAT, and POD, are crucial for maintaining the homeostasis of ROS. Under U treatment, compared with the control group, the activities of SOD, CAT, and POD in radish roots all significantly increased and then decreased with the increase in exogenous U concentration; after Cd treatment, the activities of SOD and CAT in radish roots increased significantly, while the activity of POD showed no significant change (Figure 4D–F). Under the U + Cd treatment, the decline in the activities of SOD and CAT in the radish roots was greater than that under the single U treatment. This result indicates that the presence of Cd exacerbates the inhibition by U of the antioxidant enzyme system activity in radish.

### 2.4. U and Cd Interfere with Soluble Protein and Pro Accumulation in Radish Roots

**Figure 5:**
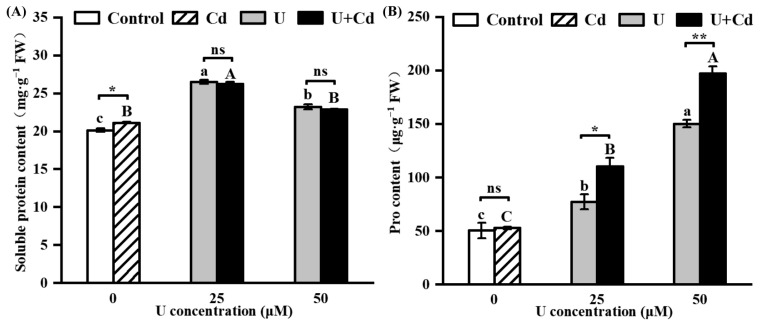
The contents of soluble protein and Pro in radish roots after 7 days of U and U + Cd stress. (**A**) Soluble protein content, (**B**) Pro content. Data shown are mean ± SD of three independent biological replicates. Different lowercase letters (a, b, c, etc.) indicate significant differences between treatments with different U concentrations at the 0.05 level (−Cd), and different uppercase letters (A, B, C, etc.) indicate significant differences between different U concentrations at the 0.05 level (+Cd) (ANOVA and LSD multiple comparison). * and ** indicate significant differences at the 0.05 and 0.01 levels between −Cd and +Cd treatments at the same U concentration, respectively (Student’s *t*-test). n.s., nonsignificant.

Figure 5 illustrates the differences in the accumulation of soluble protein and proline in radish roots under single U treatment and U + Cd combined treatment. Under single Cd treatment, the content of soluble protein in radish roots exhibited a significant increase compared to the control group, while there was no significant difference in proline content (Figure 5A,B). After U treatment and U + Cd treatment, the content of soluble protein in radish roots increased significantly, first rising and then falling with the increase in uranium concentration; there was no significant difference between single U treatment and U + Cd combined treatment (Figure 5A). After U and U + Cd treatment, the proline content was significantly higher than in the control group and increased with the increase in uranium concentration (Figure 5B). The proline content in radish roots under U25 + Cd and U50 + Cd treatments was 30.1% and 23.8% higher than that under U25 and U50 treatments, respectively (Figure 5B). This indicates that the presence of Cd promotes the accumulation of proline in radish roots under uranium stress.

### 2.5. Relative Expression Level of Genes Involved in U and Cd Transportation

**Figure 6:**
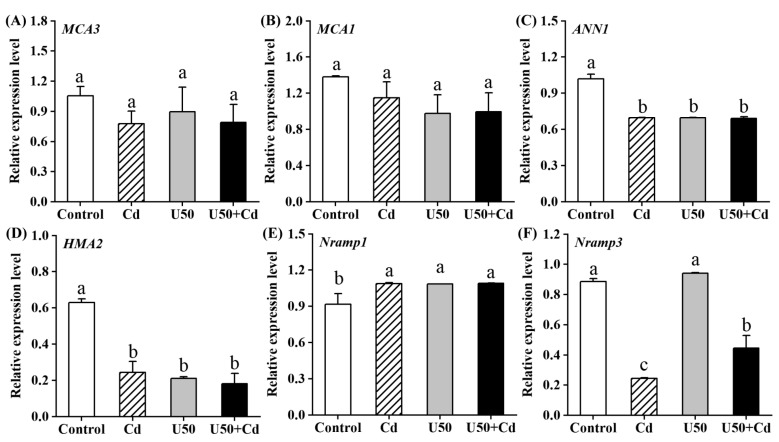
The relative expression of genes involved in U and Cd transportation after 7 days of U and U + Cd stress. (**A**) *MCA3*, the Ca^2+^-permeable mechanosensitive channel 3; (**B**) *MCA1*, the Ca^2+^-permeable mechanosensitive channel 1; (**C**) *ANN1*, annexin 1; (**D**) *HMA2*, the heavy metal P1B-ATPase 2; (**E**) *Nramp1*, the natural resistance-associated macrophage protein 1; (**F**) *Nramp3*, the natural resistance-associated macrophage protein 3. The U concentration was 50 μM, and the Cd concentration was 10 μM. Data shown are mean ± SD. Different lowercase letters (a, b, c, etc.) indicate significant differences between treatments at the 0.05 level (ANOVA and LSD multiple comparison).

Finally, we embarked on an initial exploration of the molecular mechanisms that may underlie the enhanced accumulation of U in the presence of Cd. Since the difference in U accumulation was greatest at a uranium concentration of 50 μM, the expression differences of genes related to U and Cd transport in radish were examined under control, Cd, U50, and U50 + Cd treatments (Figure 6). The results showed that the relative expression levels of *MCA1* and *MCA3* genes did not change significantly compared to the control group after U, Cd, and U + Cd treatments, while the relative expression of *ANN1* and *HMA2* genes decreased by 31.5% to 32.3% and 61.2% to 71.2%, respectively; the relative expression of the *Nramp1* gene increased by 15.6% to 15.9% (Figure 6A–E). The relative expression of these genes showed no significant differences between Cd, U50, and U50 + Cd treatments (*p* > 0.05). The relative expression level of the *Nramp3* gene was significantly lower after Cd treatment and U + Cd treatment compared to the control group, and the relative expression level of the *Nramp3* gene after U + Cd treatment was significantly higher than after single Cd treatment (Figure 6F). These results indicate that both U and Cd inhibit the expression of the *ANN1* and *HMA2* genes and promote the expression of the *Nramp1* gene; Cd has an inhibitory effect on the expression of the *Nramp3* gene, and this inhibitory effect is mitigated when U and Cd coexist.

## 3. Discussion

Both U and Cd are non-essential elements in plants, and excessive accumulation can inhibit plant growth by damaging cellular structures [41,42] and by disrupting the plant’s physiological metabolism, including the metabolism of reactive oxygen species [24,29,40]. In the present study, radish root cells died (Figure 1B), and the root biomass and root–shoot ratio were significantly inhibited (Figure 2) after U treatment at concentrations of 25 and 50 μM. These results are consistent with previous findings in *Vicia faba* [13], *Arabidopsis thaliana* [6], and *Raphanus sativus* L. [40]. The Cd treatment was less toxic to radish, potentially attributable to the reduced concentration employed, which was 10 μM.

Given that actual uranium-contaminated sites often contain associated heavy metals, particularly Cd, it is crucial to investigate the impact of these associated heavy metals on the accumulation and toxicity of U in plants. Previous studies have found that the presence of Cd increases the toxicity of U to *Arabidopsis thaliana* [24] and *Oenanthe javanica* [43] but has some mitigating effect on the toxicity of U to *Rohdea japonica* [26]. In the present study, compared with single U treatment and Cd treatment, U + Cd treatment significantly increased the enrichment of U and significantly decreased the enrichment of Cd (Figure 3), and the root cell death and the inhibition of root biomass of radish were more severe (Figure 1). This indicates that the toxic effect of increased U content is more pronounced than the mitigation provided by the decreased Cd content. Because U is not only chemically toxic but also radioactive, the toxicity of the same dose of U for plants is significantly greater than that of Cd. From an ecological restoration perspective, the presence of Cd can promote the accumulation of U in radish, which may enhance the restoration efficiency of radish in areas polluted with U. However, the reduction in biomass caused by increased U accumulation should also be carefully considered. Moreover, in complex field soil environments, particularly those amended with phosphate fertilizers or harboring active phosphate-solubilizing microorganisms, uptake of uranium and cadmium by radish is notably attenuated [44,45]. This suppression concurrently reduces Cd translocation from roots to shoots [46] and mitigates U/Cd-induced oxidative stress [47], ultimately diminishing accumulation of both metals. Therefore, conducting relevant experiments under soil culture conditions will be meaningful for further evaluating the impact of Cd presence on the U extraction efficiency from plants.

Studies have shown that uranium induces ROS bursts in various plants (such as *Nicotiana tabacum*, *Vicia faba*, *Pisum sativum* L., and *Populus cathayana*), causing peroxidation damage to cells [48]. In the present study, the O_2_^−^ production rate, H_2_O_2_ content, and peroxide damage marker MDA content were significantly increased in radish roots after U treatment (Figure 4A–C). These findings indicate that U induced oxidative damage in radish roots, corroborating the research outcomes reported by Wu et al. [40], after three days of exposure to uranium. Under U + Cd combined treatment, the rates of O_2_^−^ production, H_2_O_2_ content, and MDA content in radish roots were all higher than those under single U treatment (Figure 4A–C), indicating that the presence of Cd led to an ROS burst in radish roots under U stress. Under stress conditions, enzymatic antioxidant systems such as SOD, CAT, and POD are crucial for maintaining reactive oxygen species (ROS) homeostasis in plants. In the present study, the activities of SOD, CAT, and POD in radish were significantly increased under low concentrations of U stress, which is conducive to clearing excess ROS. However, under high concentrations of U stress, the activities of SOD and CAT were decreased. Compared with single U treatment, ROS levels in radish roots increased, and the activities of SOD, CAT, and POD were significantly reduced after U + Cd treatment, resulting in more severe peroxidation damage to cells (Figure 4D–F). This may be due to the inhibition of antioxidant enzyme gene expression caused by the presence of Cd [24]. Proline (Pro) is the most widely distributed osmotic protective substance in plants. It is able to regulate osmotic balance, remove reactive oxygen species, and maintain membrane and subcellular stability. Changes in its content can reflect the degree of stress in plants [49,50,51]. In the present study, the Pro content in radish roots increased significantly under U stress (Figure 5B), similar to the results of our previous research on *Vicia faba* [52]. Under U + Cd stress, the Pro content in radish roots was significantly higher than that under single U stress, indicating that radish might be subjected to more severe stress (Figure 5B). Overall, these results suggest that the presence of Cd intensifies U-induced oxidative stress, which may be one of the reasons that radish roots are more poisoned when Cd is present.

There are no special transporters for non-essential elements such as uranium (U) and cadmium (Cd). They are absorbed and transported by plants through competition for binding sites with elements that have similar physical and chemical properties [53,54]. The affinity and binding compatibility between ions and transporters on plant cell membranes exhibit strong dependence on ionic radius and charge density [55]. In natural environments, uranium predominantly exists as uranyl ions (UO_2_^2+^), while cadmium typically occurs as free Cd^2+^ ions. Both are divalent cations and thus can only enter plant cells through transport channels dedicated to other divalent ions [56]. Notably, UO_2_^2+^ and Cd^2+^ possess hydration radii of 0.83 Å and 0.97 Å, respectively [57,58], conferring physicochemical properties similar to calcium (Ca^2+^), manganese (Mn^2+^), and zinc (Zn^2+^) ions, which are about 0.8–1.0 Å [59,60]. Consequently, all transporters identified so far that absorb uranium or cadmium are transporters for essential nutrient elements in plants (e.g., Ca^2+^, Mn^2+^, and Fe^2+^) that also permit transport of other heavy metal ions, including Cd^2+^, Pb^2+^, and Hg^2+^ [27]. These ions universally demonstrate competitive binding and transport characteristics within these shared pathways, probably enabling them to mutually mitigate the toxicity induced by heavy metal ions [61].

Currently known transporters capable of Cd^2+^ translocation include members of the natural resistance-associated macrophage protein (*NRAMP*) family, heavy metal-transporting ATPases (*HMA*), and several other protein families [62]. In contrast, documented UO_2_^2+^ transporters are limited to only two types: the calcium channel protein annexin 1 (*ANN1*) and mid1-complementing activity (*MCA*) family proteins [28]. The *ANN1* protein, located in the plasma membrane, has been shown to facilitate the transport of U and Cd and to be involved in their uptake by plants [63,64], while *MCA1* and *MCA2*, which are proteins responsible for facilitating U translocation to shoots, are solely involved in the transport of U; there is currently no empirical evidence indicating their ability to transport Cd. The present research demonstrates that root U accumulation significantly increased under both U-only and U + Cd co-treatment conditions, whereas no U was detected in shoots (Figure 3). Notably, expression levels of *MCA1* and *MCA3* remained unaltered (*p* > 0.05). Conversely, *ANN1* expression was significantly downregulated (*p* < 0.01) following U exposure, suggesting that radish seedlings reduce U uptake through transcriptional suppression of *ANN1* (Figure 6C). *NRAMP1* is a transporter localized in the plasma membrane. In *Brassicaceae*, *NRAMP1* is homologous to *NRAMP5* in *Poaceae* and is a major transporter involved in Cd uptake in plant roots [65]. *NRAMP3*, localized in the vacuole membrane of root cells, mediates the export of manganese (Mn) and iron (Fe) from the vacuole to the cytoplasm, thereby regulating their homeostasis. It has also been shown to transport Cd [66,67]. In the present study, U + Cd treatment significantly reduced Cd accumulation in both roots and shoots compared with single treatments (*p* < 0.01). This suggests that U introduction inhibited Cd accumulation and translocation in radish seedlings (Figure 3), likely associated with decreased *Nramp1* expression and specifically elevated *Nramp3* expression (Figure 6E), which may facilitate the storage of Cd in the vacuole to mitigate its cytotoxicity. This is consistent with the findings of Thomine et al. [67] and Oomen et al. [68]. Interestingly, the gene expression level of *Nramp3* under U + Cd treatment was significantly lower than that under the control group but higher than that under single Cd treatment (Figure 6F). This could be attributed to the lower Cd content in radish under U + Cd treatment compared to the control group, allowing partial restoration of *Nramp3* gene expression to better regulate Fe and Mn homeostasis. *HMA2* mediates the xylem loading of Cd and is crucial for the transport of Cd from root to stem [69]. In the present study, the expression of *HMA2* was significantly decreased under single Cd, U, and U + Cd combined treatments compared to the control group (Figure 6D), which may reduce the translocation of Cd to the aboveground part of the plant.

While numerous studies have investigated the combined toxicological effects of U and Cd on plants, research focusing on their competitive binding at divalent cation transporter channels remains limited. In the present study, under U + Cd treatment, the expression levels of several genes implicated in the uptake of U and Cd, such as *MCA1*, *MCA3*, *ANN1*, and *Nramp1*, did not significantly differ when compared to single U or single Cd treatments (Figure 6). This suggests that changes in U and Cd accumulation are not due to the regulation of these gene expressions. Since only *MCA1*, *MCA3*, and *ANN1* have been reported to facilitate uranium uptake, there are limitations in verifying gene expression effects. However, under U + Cd treatment, the concentrations and tolerance thresholds of U and Cd in radishes did exhibit notable alterations, which could be attributed to several factors: On the one hand, the expression of other genes involved in U and Cd uptake may have been altered. On the other hand, there may be additional transporters, not limited to *ANN1*, that can mediate the uptake of both U and Cd simultaneously, with binding affinity for U being stronger than for Cd. Nevertheless, this approach warrants consideration for two reasons. First, competitive interactions among divalent ions can mitigate heavy metal toxicity. For instance, Ca^2+^ competes with Cd^2+^ for binding sites, thereby reducing Cd accumulation [70], as also observed with lead (Pb^2+^) and mercury (Hg^2+^) [71]. Second, recent studies reveal that plants exhibit preferential uptake of U over certain divalent ions (e.g., Fe^2+^ and Ca^2+^), disrupting ion homeostasis. U competitively displaces these ions at binding sites, and U-induced Fe release further triggers oxidative stress in plants [72]. Therefore, future research should investigate the uranium transport capacity of a broader range of transporters as well as their binding preferences for U and Cd.

## 4. Materials and Methods

### 4.1. Experimental Materials and Plant Culture

Radish (*Raphanus sativus* L.) seeds, obtained from the Chengdu seed market, were utilized in the present study. Uranyl nitrate [UO_2_(NO_3_)_2_·6H_2_O] and cadmium chloride (CdCl_2_·5H_2_O) served as exogenous pollutants. The analytically pure chemicals used in the experiments were purchased from Chengdu Chron Chemical Co., Ltd., Chengdu, China.

The seeds were randomly selected and sterilized using 70% alcohol. They were then rinsed three times with distilled water, soaked for a duration of 24 h, and subsequently transferred to a petri dish (diameter = 9 cm) lined with a layer of filter paper. Finally, the seeds were placed in a germination chamber set at a temperature of 28 °C to facilitate sprouting. The seeds were transferred to group culture flasks for cultivation when the root length reached approximately 0.5 cm. UO_2_^2+^ readily combines with phosphate, leading to precipitation and subsequently reducing its bioavailability. Consequently, in this experiment, radishes were treated with a modified Hoagland nutrient solution based on our previous research [73], which lacked phosphorus and contained UO_2_^2+^ at pH 5.5. Each culture flask contained 200 mL, replaced every three days to maintain optimal conditions [74]. To avert phosphorus deficiency in the plants, foliar fertilization was administered utilizing a 0.1 mmol·L^−1^ phosphate solution, which was sprayed consistently at regular intervals every day. The culture conditions were maintained as follows: a temperature of 20–25 °C, an illumination intensity of 3500 lux, and a photoperiod of 12 h of light alternating with 12 h of darkness. The specific culture conditions and Hoagland solution composition are shown in Table S1.

### 4.2. Experimental Design

As illustrated in Table 1, the nutrient solution lacking both U and Cd served as a blank control. The mean concentration of Cd in the agricultural soil surrounding the U tailings was approximately 0.64 mg·kg^−1^ (5.69 μM) [75]. Consequently, the Cd concentration was adjusted to 10 μM for the experimental setup. The U concentration in the soil of the U tailings varied approximately from 5.2 to 48.1 mg·kg^−1^ (from 21.85 to 202.08 μM) [38]. Notably, the maximum U concentration that plants could absorb did not surpass 100 μM [76]. Consequently, the U concentrations were adjusted to 25 and 50 μM for the present study. When the second true leaves of the radish seedlings turned green about 21 days after planting, they were subjected to various concentrations of U alone and U + Cd composite treatment solutions (Table 1). Daily phosphate spraying was maintained throughout the experiment. After a treatment duration of 7 days under consistent cultivation conditions, samples were collected and relevant physiological parameters were measured.

### 4.3. Experimental Methods

#### 4.3.1. Detection of Root Activity

After 7 days of treatment, the plants were photographed. The viability of root cells was assessed through FDA/PI staining (FDA: 5 g·L^−1^ and PI: 50 mg·L^−1^) after 3, 5, and 7 days of treatment. The root tips (0.5 cm) were excised and rinsed with double-distilled water (ddH_2_O). Subsequently, they were stained in the dark for a duration of 10 min. After staining, the roots were rinsed again with ddH_2_O and then imaged using fluorescence microscopy (Eclipse 55i, Nikon Co., Ltd., Tokyo, Japan) at a magnification of 50×. The excitation/emission wavelengths for FDA were 488/515 nm, and those for PI were 543/585 nm [13].

#### 4.3.2. Biomass Determination

Radish seedlings were meticulously washed with 20 mM ethylenediaminetetraacetic acid (EDTA) to remove surface contaminants, followed by repeated rinsing with ddH_2_O to ensure thorough cleanliness. Subsequently, the root and aboveground portions were separately dried at 80 °C until they reached a consistent weight. The dry biomass was then measured, and the root–shoot ratio was determined [77]. Each experiment was replicated three times to ensure reliability of the results.

#### 4.3.3. Determination of Physiological Parameters of the Radish Roots

Radish roots were carefully washed with ultrapure water. Subsequently, three fresh samples, each precisely weighing 1.0 g, were swiftly prepared to ascertain the rate of O_2_^−^ production by the TCA method and to determine H_2_O_2_ content by the KI method [78]. Additionally, the content of malondialdehyde (MDA) was measured using the thiobarbituric acid (TBA) method [79], while the proline (Pro) content was quantified by the acid ninhydrin method [52].

A 0.2 g sample of roots to be tested was weighed and placed in 5 mL of 0.05 M phosphate buffer (pH 7.8) along with a small quantity of quartz sand. This mixture was then ground to homogenization in an ice bath. Following this, the solution was centrifuged at 8000 r·min^−1^ for 20 min at 4 °C. The supernatant, referred to as the enzyme solution, was carefully transferred to test tubes for storage. Subsequently, the activity of superoxide dismutase (SOD) was determined using the nitrotetrazolium chloride (NBT) method, peroxidase (POD) activity was determined by the guaiacol method, catalase (CAT) activity was measured through a spectrophotometric method, and the content of soluble protein was measured by the Coomassie brilliant blue method [73]. Each experimental index was replicated three times to ensure the reliability and consistency of the results.

The detailed information of the above method has been clearly stated in the Supplementary Materials.

#### 4.3.4. U and Cd Content Detection

The collected radish seedlings were partitioned into underground (root) and aboveground (stem and leaf) portions. These parts were initially subjected to a high temperature of 105 °C for approximately 30 min for green removing, then baked at 80 °C until a consistent weight was achieved. The dried samples were then crushed using a mortar and pestle. Approximately 1.0 g of each sample from both the underground and aboveground parts was weighed into a 150 mL conical flask. Subsequently, 15 mL of an acid solution composed of HNO_3_ and HClO_4_ in a 3:1 ratio (*v*/*v*) was added to ablate the samples. Upon complete ablation, the samples were adjusted to a final volume of 50 mL. The Cd content was determined using flame atomic absorption spectrophotometry [80]. The U content was determined by following the method described by Khan et al. [81]. Each measurement was repeated three times to ensure accuracy and reliability.

#### 4.3.5. Analysis of Gene Expression

After a seven-day treatment with various solutions, the roots were washed three times with ddH_2_O and immediately stored in liquid nitrogen. Total RNAs were extracted from the roots using Trizol reagent. The total RNAs were then reverse-transcribed into cDNA. Gene-specific primers were designed using Primer Premier5, and the sequences for these primers are listed in Table 2. The expression levels of each gene were measured by RT-PCR using *ACT7* as the reference gene. The Image J software (1.8.0, National Institutes of Health, Bethesda, MD, USA) was used to determine the grayscale values of each electrophoretic band. The relative expression levels of these genes were calculated by dividing the grayscale value of their bands by that of the *ACT7* band [75]. For each biological replicate, samples from three individual plants were pooled to ensure representativeness and minimize variability. A total of three such biological replicates were rigorously analyzed to assess gene expression, ensuring the reliability and robustness of the results.

### 4.4. Statistical Analysis

Statistical analyses were performed using Microsoft Excel 2021 (Microsoft Inc., Redmond, WA, USA) and SPSS 26.0 software (SPSS Inc., Chicago, IL, USA) to collect and analyze data. The Shapiro–Wilk test assessed data normality (*p* > 0.05), and a subsequent significance test was performed. For groups that were 2 (N = 2), a t-test was performed. For groups (N) that were greater than 2 (N > 2), one-way analysis of variance (ANOVA) and least significant difference (LSD) tests were performed. Data are expressed as mean ± SD. The results were visualized using Origin 2024 (Origin Lab. Corporation, Northampton, MA, USA), while Adobe Illustrator CC 2019 (Adobe Systems Inc., San Jose, CA, USA) was employed to enhance the formatting and typography of the images.

## 5. Conclusions

The present study revealed that the presence of Cd significantly amplified the accumulation and toxicity of U in radish. U + Cd treatment caused more severe cell death and more obvious biomass reduction in radish compared with single U treatment. The stress induced by U led to an escalation in the levels of reactive oxygen species (ROS) in the radish roots, inflicting substantial peroxidation damage on the cells. This damage was notably more severe under the U + Cd combined treatment. The study also scrutinized the relative expression levels of major genes associated with the absorption and transport of U and Cd in plants. Interestingly, under the U + Cd combined treatment, the expression levels of U transport-related genes (*MCA1*, *MCA3*, *ANN1*, and *Nramp1*) exhibited no significant differences compared to single U or Cd treatments. However, the inhibitory effect of the Cd transport-related gene *NRAMP3* was attenuated, suggesting that U exacerbates toxicity by promoting Cd transport, which necessitates further in-depth research in the future.

## Figures and Tables

**Table 1:** Experimental design.

Treatments	U0	U25	U50	Cd	U25 + Cd	U50 + Cd
U concentration (μM)	0	25	50	0	25	50
Cd concentration (μM)	0	0	0	10	10	10

**Table 2:** Specific primers for genes involved in U and Cd transport mechanisms.

Gene Name	Forward Primer (5′→3′)	Reverse Primer (5′→3′)
*ACT7*	GATGGGTCAGAAAGATGC	CTGTTGGCTTTAGGGTTA
*MCA1*	TCCGTATCGTATCCCTAACA	GGAGCCGTGACCAGAGT
*MCA3*	GTCCTTGATTTACCCTCTG	GTTCCACCATTTGTTCCT
*ANN1*	AATGCTACTTTCAATCGCTAC	GCTGTTCCTCCTCTGATACTC
*Nramp1*	CAATCTGGAGCACAATACA	CAGCAACAACCCATAGC
*Nramp3*	AATCGGTCCTTTGTATCAGA	ATGCCACGAGCAATCAG
*HMA2*	GTAATAACCGTGGGAGC	GATTGGAGCCATTCAGC

## Data Availability

Data available upon request.

## Supplementary Materials

The following supporting information can be downloaded at https://www.mdpi.com/article/10.3390/plants14172711/s1, Table S1: Cultural Conditions.
