# Supplementary material for: Effects of Cadmium on the Accumulation and Phytotoxicity of Uranium in Radish (Raphanus sativus L.) Seedlings
Source: Plants (Basel). 2025 Sep 1;14(17):2711. doi: 10.3390/plants14172711 (PMC12430486; doi:10.3390/plants14172711)
Supplement: Supplementary file 1 [file plants-14-02711-s001.zip › plants-3812588-supplementary.pdf]

Supplementary Information For

Effects of cadmium on the accumulation and phytotoxicity of uranium in radish (*Raphanus sativus* L.) seedlings

Xin-Peng Guo [1], Xi Chen [2], Chun-Xia Tu [3], Yu-Meng Fan [1], Ming-Xuan Wang [1], Zheng-Qin Zhao [1], Shi-Yi Yang [1], Lan-Lan Cui [1], Guo Wu [1], Jin-Long Lai [4] and Qun Li [1*]**

[1] College of Life Science, Sichuan Normal University, Chengdu, 610101, China; shixinpu19610613@163.com (X-P.G.); 18328640034@163.com (Y-M.F.); 13994652071@163.com (M-X.W.); 13778309360@163.com (Z-q. Z.); 15328972277@163.com (S-Y.Y.); cuiyounian@gmail.com (C-L.L.); wuguoyk@sicnu.edu.cn (G.W.)

[2] College of Resources and Environmental Sciences, Nanjing Agricultural University, Nanjing 210095, China; chenx_19981008@163.com (X.C.)

[3] State Key Laboratory of Cellular Stress Biology, Xiamen University, Xiamen, 361000, China; tuchunxia@stu.xmu.edu.cn (C-X.T.)

[4] Engineering Research Center of Biomass Materials, Ministry of Education, Southwest University of Science and Technology, Mianyang, 621010, China;

* Correspondence: laijinlongswust@163.com (J-L.L.)；liqun01234@163.com (Q.L.);
Tel. /Fax: +86-28-84480656

Supporting Material and Methods

Table S1. Culture conditions

Indicators	Conditions
Hongland Nutrient Solution	1 mM KNO_3<br>1 mM $Ca(NO_3)_2·4H_2O$<br>0.4 mM $MgSO_4·7H_2O$<br>0.2 mM NH_4NO_3<br>4 μm EDTA-Fe<br>4.5 μM $MnCl_2·4H_2O$<br>0.38 μM $ZnSO_4$<br>0.1 μM $CuSO_4·5H_2O$<br>0.25 μM $H_2MoO_4·4H_2O$<br>$UO_2(NO_3)_2·6H_2O$ according to experimental design<br>$CdCl_2$ according to experimental design<br>pH 5.5
Temperature	25 - 30 ℃
Light intensity	3500 lux, 12 h light/12 h dark cycle
Related Humidity	70 - 80 %

Methods for Determination of physiological parameters of the radish roots

Malondialdehyde (MDA) Content

Weigh 0.5 g of Pak Choi leaves and homogenize them in increments with 3 mL of 10% trichloroacetic acid (TCA). Transfer the homogenate to a centrifuge tube and centrifuge at 4,000 rpm for 10 minutes. Collect 2 mL of the supernatant and mix it with 2 mL of 0.6% thiobarbituric acid (TBA, prepared in 10% TCA). Vortex for 30 seconds after sealing and heat in a boiling water bath for 15 minutes. After cooling, measure the absorbance at 450 nm, 532 nm, and 600 nm. Calculate the MDA content (μmol/g) using the following formula:

$$\text{MDA content (μmol/g)} = \frac{(6.45\times(A_{532}\text{-}A_{600})\text{-}0.56\times A_{450})\times 0.004\times 3}{m\times 2\times 1000}$$

Where "*m*" represents the sample mass (g), A_{459}, A_{532} and A_{600} represent the absorbance of the sample at 450, 532 and 600 nm, respectively.

Proline content

The plants were washed 3 times with ddH_2O and dried with absorbent paper, ground thoroughly in 3% sulfosalicylic acid on ice, and extracted in a water bath at 100 °C for 10 min. After centrifugation at 3000 r min^{-1} for 10 min, 2 mL of the upper clarified solution was taken, 3 mL ninhydrin hydrate and 2 mL glacial acetic acid were added and then boiled in a water bath for 45 min. After cooling in an ice bath, 5 mL toluene was added, mixed thoroughly, and allowed to stand and layer. The upper phase was detected at 520 nm

Dismutase (SOD), peroxidase (POD), catalase (CAT) activities

In detail, 0.2 g samples were placed in a mortar with quartz sand, and 5 mL of PBS (50 mM, pH 7.8) was added. After grinding into the homogenate on ice, the mixture was centrifugedat 7000 rcf and 4 ◦C for 20 min. The activities of superoxide dismutase (SOD), peroxidase(POD), and catalase (CAT) in the supernatant were determined by the tetrazolium blue(NBT) photoreduction method, ultraviolet colorimetry, and guaiacol method, respectively. Superoxide dismutase (SOD) activity was determined spectrophotometrically at 560 nm,with one unit of enzyme activity (U) defined as the amount of enzyme required to inhibitthe photochemical reduction of 50% NBT, representing one unit of fresh weight (U·g−1 FW). Catalase (CAT) activity was determined spectrophotometrically at 240 nm, with one unit ofenzyme activity (U) defined as the amount of enzyme required to increase the absorbance by 0.01 per minute per gram of fresh weight ($U \cdot g^{-1}$ FW). Peroxidase (POD) activity was determined spectrophotometrically at 470 nm, with one unit of enzyme activity (U) defined as the amount of enzyme required to increase the absorbance by 0.1 per minute per gram of fresh weight ($U \cdot g^{-1}$ FW).

Soluble protein content

In detail, the samples were homogenized with 5 mL of 0.05 M phosphate buffer (pH 7.8). The homogenates were then centrifuged at 4 °C and 8000 rpm for 20 min. A volume of 20 μL of the supernatant was collected. Subsequently, 3 mL of a 0.1 g/L Coomassie Brilliant Blue G-250 solution was added, and the mixture was allowed to stand for 5 min. The absorbance was measured at 595 nm.
